# Association of Patient Comorbidities With Treatment Regret Among Patients With Localized Prostate Cancer – Results From a Population-Based Cohort

**DOI:** 10.1016/j.prro.2025.07.007

**Published:** 2025-08-31

**Authors:** Rahul D. Mali, Ying Cao, Aaron J. Katz, Katelyn Kane, Yahui Xie, Deborah S. Usinger, Xinglei Shen, Ronald C. Chen

**Affiliations:** a Department of Radiation Oncology, University of Kansas Medical Center, Kansas City, Kansas; b Department of Population Health, University of Kansas Medical Center, Kansas City, Kansas; c Lineberger Comprehensive Cancer Center, University of North Carolina at Chapel Hill, Chapel Hill, North Carolina

## Abstract

**Purpose:**

Decision regret is a well-established negative outcome in prostate cancer. We hypothesized that baseline comorbidities, which impact treatment tolerability, are associated with regret.

**Methods and Materials:**

In a prospective, population-based cohort of patients with prostate cancer, patient-reported regret was assessed at 12 months after treatment using a validated measure. Comorbidities were assessed using medical record abstraction and scored using the validated National Cancer Institute Comorbidity Index. Multivariable logistic regression was used to assess the association between the comorbidity score and regret, accounting for treatment-related symptoms, treatment received, and sociodemographic measures.

**Results:**

This was a diverse cohort, comprising 25.3% Black patients and 24.2% living in rural areas. A total of 108 out of 981 patients (11%) reported regret. In multivariable analysis, comorbidity score (odds ratio [OR], 1.58; *p* < .05), not being married (OR, 1.72; *p* = .04), worsening of bowel symptoms (OR, 2.12; *p* < .01), and worsening of urinary obstruction/irritation (OR, 1.60; *p* = .05) were associated with decision regret. In addition, radiation therapy was associated with less regret compared with radical prostatectomy (OR, 0.48; *p* = .015).

**Conclusions:**

Among men with localized prostate cancer, baseline comorbidity burden was associated with increased decision regret. These results illustrate the importance of assessing baseline comorbidities and incorporating their consideration into the treatment decision-making process, ensuring that patients have realistic expectations and make informed decisions.

## Introduction

Prostate cancer is the second most commonly diagnosed cancer among men worldwide.^1^ Management options are guided by prognostic factors, including prostate-specific antigen level, clinical TNM stage, and the Gleason score, in addition to quality of life (QOL) and patient preference considerations.^2^ Standard approaches include active surveillance, surgery, and radiation therapy, which are associated with different impacts on QOL.^3–5^

The decision-making process for a patient diagnosed with prostate cancer is complex, and patients experience varying degrees of decision regret after treatment.^6–9^ Decision-related regret is a negative emotion associated with thinking about a past or future choice^10,11^ and is an important patient-centered outcome.^12^ Decision regret adversely impacts patients’ QOL and causes emotional distress.^13^

Previous literature has demonstrated that decision regret in prostate cancer is associated with patient age and race, socioeconomic status, and health-related QOL.^14–18^ Because a patient’s baseline comorbidity burden impacts his ability to tolerate prostate cancer treatment and, therefore, treatment-related side effects, we hypothesized that there is an association between comorbidity and decision regret. If indeed this association is demonstrated, it would fill a current knowledge gap in the literature and provide new information that can be used in clinical counseling of patients with newly diagnosed prostate cancer. This is the primary goal of the current study.

## Methods and Materials

### Data source and study population

The North Carolina Prostate Cancer Comparative Effectiveness & Survivorship Study is a prospective, population-based cohort of patients with newly diagnosed localized prostate cancer enrolled throughout North Carolina in collaboration with the Rapid Case Ascertainment system of the North Carolina Central Cancer Registry. The Rapid Case Ascertainment system proactively identified patients with newly diagnosed prostate cancer in all 100 North Carolina counties for this study, thus creating a population-based observational cohort. Patients were enrolled from January 1, 2011, to June 30, 2013. Unique to this cohort, all patients were enrolled prior to treatment and followed prospectively, with the collection and abstraction of patient-reported outcomes and medical records. Details related to study methodology have been described previously.^19^ This study was approved by the University of North Carolina institutional review board, including consent to obtain records. Study data were collected and managed using REDCap electronic data capture tools hosted at the University of North Carolina at Chapel Hill.^20,21^ Written informed consent was obtained from all patients, and data were deidentified for analysis.^22^

### Study measures

Patient-reported treatment regret was measured at 1 year after treatment using a validated instrument developed by Clark et al,^13^ specific for prostate cancer. Participants were asked 2 questions: whether they would have been better off with a different treatment (definitely false, somewhat false, neither true nor false, somewhat true, or definitely true) and the amount of time spent wishing they could change their mind about the treatment (none of the time, rarely, neither a little nor a lot of the time, some of the time, or all of the time). This treatment regret scale was scored from 0 to 100, with higher scores indicating greater regret. The scale was then dichotomized: patients with a regret score of at least 40 were classified as having regret based on previously published methods by Clark et al.^8,12,23^

Baseline medical records were collected on study enrollment, and comorbid conditions were abstracted soon after receipt of records. Specific conditions abstracted included myocardial infarction, congestive heart failure, renal disease, peptic ulcer disease, peripheral vascular disease, cerebrovascular disease, chronic obstructive pulmonary disease (COPD), rheumatologic disorders, liver disease, diabetes, dementia, paralysis, and HIV/AIDS – which together were used to calculate the National Cancer Institute (NCI) Comorbidity Index,^24^ a validated measure of comorbidity burden in patients with prostate cancer.^25^

Covariates included individual-level sociodemographic information (race, marital status, and household income), which was collected by patient report. To account for treatment-related side effects, which can impact decision regret in patients with prostate cancer, the validated Prostate Cancer Symptom Indices (PCSI)^26^ was assessed at baseline and 12 months after treatment. The PCSI has 4 domains: urinary obstruction and irritation (5 items), urinary incontinence (3 items), sexual dysfunction (5 items), and bowel problems (6 items). Results of these questions are then calculated into 3 levels of function (normal, intermediate, or poor) for each domain using a methodology validated for the PCSI.^27,28^

### Statistical analysis

The association between baseline comorbidity burden (NCI Comorbidity Index) and treatment regret was assessed using logistic regression in univariate and multivariable models. Multivariable models accounted for patient age, race, marital status, urban or rural residence (using rural-urban continuum codes, where 1 to 3 were defined as urban and 4–9 as rural), household income, National Comprehensive Cancer Network risk group at diagnosis, initial treatment modality, and patient-reported symptoms (PCSI). The NCI Comorbidity Index was dichotomized into 0 vs >0 (with >0 indicating higher comorbidity burden), consistent with established methodology.^29^ PCSI results were categorized into patients who experienced a worsening of symptoms in each of the 4 domains (ie, baseline normal function and 12-month intermediate or poor function, or baseline intermediate function and 12-month poor function) or no worsening.

All tests used a 2-sided p value of <.05 for denoting statistical significance. All statistical analyses were performed using SAS statistical software, version 9.4 (SAS Institute).

## Results

Among 981 patients, the mean age at the time of prostate cancer diagnosis was 64.2 years (Table 1). This population-based cohort was diverse, comprising 25.3% Black patients and 24.2% living in rural areas.

Table 2 summarizes the prevalence of individual comorbid conditions at the time of diagnosis. Overall, 38.0% of the cohort had a calculated NCI Comorbidity Index score of >0. Table 3 shows that at 1 year after treatment, 37.3% of patients reported worsened urinary incontinence compared with baseline, 26.2% worsened urinary obstruction and irritation, 26.2% worsened bowel problems, and 38.6% worsened sexual dysfunction.

A total of 11.0% (N = 108) of prostate cancer survivors reported decisional regret at 1 year after treatment. In the univariate analysis (Table 4), decision regret was significantly higher in patients with a higher comorbidity, aged >=65 years, Black, not married, those who received radical prostatectomy (compared with active surveillance and radiation therapy), and those who had worsened bowel problems. In further exploring the association between individual comorbid conditions and regret, cerebrovascular disease (p = .01) and COPD (p = .03) were statistically significant findings (Table 4).

In multivariable analysis (Table 5), higher comorbidity (NCI Comorbidity Index score > 0 vs 0; odds ratio [OR], 1.58; p < .05) remained statistically significantly associated with regret. In addition, worsened bowel problems (OR, 2.12; p < .01) and not being married (OR, 1.72; p = .04) were also associated with regret. Worsened urinary obstruction and irritation (p = .05) was of borderline significance. The treatment variable overall (p = .07) was of borderline significance; however, the difference between radical prostatectomy and radiation therapy (OR, 0.48; 95% CI, 0.26-.87; p = .015) was statistically significant.

## Discussion

In this study of a sociodemographically diverse cohort, reported decisional regret at 1 year after treatment. Supporting our hypothesis, patients who had a higher comorbidity burden at baseline were more likely to have regret. In addition, treatment-related symptoms (worsening of bowel problems and potentially worsening of urinary obstruction and irritation) were also associated with regret, and radical prostatectomy was associated with more regret compared with radiation therapy.

Our finding that the vast majority of patients with prostate cancer do not have regret is consistent with prior studies.^8,12,17^ Wallis et al^12^ reported a 13% treatment regret at 5 years in the prospective population-based Comparative Effectiveness Analysis of Surgery and Radiation study, which included patients with prostate cancer from 5 Surveillance, Epidemiology, and End Results registries. Hoffman et al^8^ have previously shown a similar rate of treatment regret in 14.6% of men with localized prostate cancer in the large population-based Prostate Cancer Outcomes Study cohort at 15 years after diagnosis.

To our knowledge, this is the first study to find an association using multivariable analysis between a cancer patient’s comorbidity burden and treatment regret, adjusting for relevant covariates including age, race, and treatment received. This finding lends support to the importance of routine assessment of baseline comorbidity burden and incorporating its consideration into the treatment decision-making process. Indeed, the overtreatment of some patients with early prostate cancer is a well-recognized problem.^30^ Greater comorbidity burden may increase treatment regret among patients with prostate cancer through several interconnected mechanisms. Patients with a high comorbidity burden are more likely to experience treatment-related side effects^31,32^ and have slower recovery, and are therefore less likely to benefit from aggressive treatment and more likely to be harmed by it.^33,34^ Comorbidities result in lower functional status at baseline, which has been associated with greater dissatisfaction after treatment in patients with prostate cancer.^35^ Previous studies have also shown that a higher comorbidity burden is correlated with poor social support, which is a significant predictor of decision regret.^36,37^

Our additional findings that treatment-related symptoms (specifically urinary and bowel symptoms) and treatment received (radical prostatectomy more than radiation therapy) were associated with regret are similarly clinically relevant. Shared decision-making, after evaluating comorbid conditions, can potentially reduce decision regret by facilitating informed decisions and providing realistic expectations regarding treatment outcomes.^38^ Univariate analysis found statistically significant associations between regret and cerebrovascular disease and COPD; patients with these comorbidities may have a more limited life expectancy and higher risks of treatment-related side effects than healthier patients, so discussions related to potential benefits versus harms of aggressive treatment are needed. Our findings from the multivariable model that worsened urinary obstruction/ irritation and bowel problems were associated with more regret highlight the importance of pretreatment counseling to ensure that patients understand potential side effects that can occur.

Our study has several limitations. This analysis only assessed regret at 1 year after treatment. Although treatment-related side effects often recover by this time,^39^ some patients have residual symptoms that can impact regret. Therefore, we controlled for this in the multivariable model. However, regret that might have occurred earlier because of acute side effects after surgery or radiation therapy was not captured. Another limitation was that comorbidities were identified through medical record abstraction, which might impact the ability to accurately capture the prevalence and severity of comorbid conditions. However, in our prior methodologic work using data from the North Carolina Prostate Cancer Comparative Effectiveness & Survivorship Study cohort, we found that medical record abstraction versus patient reporting of comorbid conditions yielded similar results.^22^ In addition, because only 11% of the cohort expressed regret, statistical power was somewhat limited. However, despite this, the study was able to demonstrate an association between baseline comorbidity and regret, informing our primary hypothesis. There are also multiple strengths to this study, including a population-based and diverse cohort, individually collected sociodemographic data, prospectively evaluated patient-reported outcomes (including baseline QOL before treatment), and the use of validated assessments throughout this study.

## Conclusions

Among men with localized prostate cancer, baseline comorbidity is associated with treatment regret. Further, radical prostatectomy is associated with more regret than radiation therapy. These results illustrate the importance of assessing baseline comorbidities and incorporating their consideration into the treatment decision-making process, ensuring that patients have realistic expectations and make informed decisions.

## Figures and Tables

**Table 1:** Patient demographics and baseline characteristics

Variable	No. of patients (N = 981)	% of Patients

Age (y) at diagnosis, mean (SD)	64.2 (7.44)	
<65	489	49.8
≥65	492	50.2
Race		
White	733	74.7
Black	248	25.3
Marital status		
Married	793	80.8
Not married	188	19.2
Geographic location of residence		
Urban	744	75.8
Rural	237	24.2
Household income, $/y		
<40,000	339	34.6
40,000–90,000	398	40.6
>90,000	211	21.5
Refused	33	3.4
NCCN prostate cancer risk group*		
Low	484	49.5
Intermediate	363	37.1
High	131	13.4
Treatment groups		
Radical prostatectomy	393	40.1
Active surveillance	234	23.9
Radiation therapy	278	28.3
Others^†^	76	7.7
PCSI urinary incontinence		
Normal	485	49.4
Intermediate	259	26.4
Poor	231	23.6
Missing	6	0.6
PCSI urinary obstruction/irritation		
Normal	262	26.7
Intermediate	348	35.5
Poor	362	36.9
Missing	9	0.9
PCSI bowel problems		
Normal	379	38.6
Intermediate	439	44.8
Poor	161	16.4
Missing	2	0.2
PCSI sexual dysfunction		
Normal	83	8.5
Intermediate	179	18.2
Poor	700	71.4
Missing	19	1.9

*Abbreviations:* HIFU, high-intensity focused ultrasound; NCCN, National Comprehensive Cancer Network; PCSI, Prostate Cancer Symptom Indices.

* 3 patients had missing information about NCCN risk categories.

† Others included HIFU, cryoablation, and hormone treatment alone.

**Table 2:** Patient baseline comorbidities

Variable	No. of Patients (N = 981)	% of the Patients

Individual comorbidities		
Myocardial infarction	66	6.7
Congestive heart failure	25	2.5
PVD	30	3.1
Renal disease (moderate/severe)	47	4.8
AIDS	0	0
Peptic ulcer disease	36	3.7
Cerebrovascular disease	57	5.8
COPD	70	7.1
Rheumatologic disorders	2	0.2
Liver disease (moderate/severe)	2	0.2
Diabetes	209	21.3
Dementia	2	0.2
Paralysis	2	0.2
NCI Comorbidity Index score		
0	608	62.0
>0	373	38.0

*Abbreviations:* COPD, chronic pulmonary obstructive disease; NCI, National Cancer Institute; PVD, peripheral vascular disease.

**Table 3:** Change in patient-reported symptoms at 1 year after treatment compared with baseline

Variable	N	%

Urinary incontinence*		
No worsening	615	62.7
Worsened	366	37.3
Urinary obstruction/irritation*		
No worsening	711	73.8
Worsened	253	26.2
Bowel problems*		
No worsening	720	73.8
Worsened	255	26.2
Sexual dysfunction*		
No worsening	584	61.4
Worsened	367	38.6

* Missing data, ie, unable to calculate domain scores (response to 1 or more questions was missing or unknown) for urinary obstruction/irritation (n = 17), urinary incontinence (n = 0), bowel dysfunction (n = 6), and sexual dysfunction (n = 30).

**Table 4:** Univariate logistic regression analysis of factors associated with decision regret

Variable	Odds ratio (95% CI)	*P* value

NCI Comorbidity Index score (ref: 0)		
>0	1.60 (1.07–2.39)	.02
Age at diagnosis (ref: <65 y)		
≥65 y	0.65 (0.43–0.98)	.04
Race (ref: White)		
Black	1.80 (1.18– 2.74)	.01
Marital status (ref: married)		
Not married	2.26 (1.45–3.50)	<.01
Geographic location (ref: urban)		
Rural	0.99 (0.62–1.59)	.98
Household income, $/y (ref: >90,000)		
<40,000	1.19 (0.67–2.11)	.32
40,000–90,000	1.51 (0.85 –2.66)	-
Refused	1.01 (0.28–3.62)	-
NCCN risk groups (ref: low)		
Intermediate	0.91 (0.58–1.43)	.08
High	1.71 (1.00–2.95)	-
Treatment groups (ref: radical prostatectomy)		
Active surveillance	0.51 (0.29–0.90)	.02
Radiation therapy	0.55 (0.33–0.93)	-
Others*	1.15 (0.58–2.27)	-
PCSI (ref: no worsening)		
Sexual dysfunction		
Worsened	0.87 (0.57–1.32)	.50
Urinary obstruction and irritation		
Worsened	1.49 (0.95–2.31)	.07
Urinary incontinence		
Worsened	1.46 (0.97–2.18)	.07
Bowel problems		
Worsened	2.17 (1.44–3.29)	<.01
Individual comorbid conditions		
Myocardial infarction	0.70 (0.28–1.73)	.44
Congestive heart failure	0.48 (0.09–2.63)	.40
Renal disease (moderate/severe)	1.52 (0.67–3.43)	.31
PVD	1.77 (0.68–4.61)	.24
Cerebrovascular disease	2.36 (1.21–4.59)	.01
COPD	2.01 (1.07–3.78)	.03
Rheumatological disorders	1.60 (0.04–66.60)	.14
Liver disease (moderate/severe)	8.11 (0.50–130.59)	.62
Diabetes	1.08 (0.67–1.74)	.76
Dementia	8.11 (0.50–130.59)	.14
Peptic ulcer disease	0.83 (0.27–2.59)	.75
Paralysis	1.60 (0.04–66.60)	.80

*Abbreviations:* COPD, chronic pulmonary obstructive disease; NCCN, National Comprehensive Cancer Network; NCI, National Cancer Institute; PCSI, Prostate Cancer Symptom Indices; PVD, peripheral vascular disease; Ref, reference.

* Others included high-intensity focused ultrasound (HIFU), cryoablation, and hormone treatment alone.

**Table 5:** Multivariable logistic regression analysis of factors associated with decision regret

Variable	Odds ratio (95% CI)	*P* value

NCI Comorbidity Index score (ref: 0)		
>0	1.58 (1.00–2.48)	<.05
Age at diagnosis (ref: <65 y)		
≥65 y	0.69 (0.43–1.10)	.12
Race (ref: White)		
Black	1.40 (0.83–2.34)	.20
Marital status (ref: married)		
Not married	1.72 (1.02–2.89)	.04
Geographic location (ref: urban)		
Rural	0.95 (0.57–1.58)	.83
Household income, $/y (ref: >90,000)		
<40,000	0.96 (0.48–1.94)	.88
40,000–90,000	1.10 (0.59–2.04)	-
NCCN risk groups (ref: low)		
Intermediate	0.85 (0.51–1.42)	.46
High	1.29 (0.69–2.40)	-
Treatment groups (ref: radical prostatectomy)		
Active surveillance	0.59 (0.31–1.15)	.07
Radiation therapy	0.48 (0.26–0.87)	-
Others*	0.98 (0.44–2.21)	-
PCSI (ref: no worsening)		
Sexual dysfunction		
Worsened	0.67 (0.40–1.10)	.11
Urinary obstruction and irritation		
Worsened	1.60 (1.00–2.57)	.05
Urinary incontinence		
Worsened	1.08 (0.67–1.72)	.76
Bowel problems		
Worsened	2.12 (1.33–3.36)	<.01

*Abbreviations:* HIFU, high-intensity focused ultrasound; NCCN, National Comprehensive Cancer Network; NCI, National Cancer Institute; PCSI, Prostate Cancer Symptom Index; Ref, reference.

* Others included HIFU, cryoablation, and hormone treatment alone.
